# In Vitro and In Vivo Leishmanicidal Activity of *Astronium fraxinifolium* (Schott) and *Plectranthus amboinicus* (Lour.) Spreng against *Leishmania (Viannia) braziliensis*

**DOI:** 10.1155/2014/848293

**Published:** 2014-04-16

**Authors:** Silvio César Gomes de Lima, Maria Jania Teixeira, José Evaldo Gonçalves Lopes Júnior, Selene Maia de Morais, Alba Fabiola Torres, Milena Aguiar Braga, Raphael Oliveira Rodrigues, Gilvandete Maria Pinheiro Santiago, Alice Costa Martins, Aparecida Tiemi Nagao-Dias

**Affiliations:** ^1^Departamento de Análises Clínicas e Toxicológicas, Faculdade de Farmácia, Universidade Federal do Ceará (UFC), Rua Capitão Francisco Pedro 1210, 60430-370 Fortaleza, CE, Brazil; ^2^Departamento de Patologia e Medicina Legal, Faculdade de Medicina, UFC, Rua Monsenhor Furtado S/N, 60430-350 Fortaleza, CE, Brazil; ^3^Departamento de Química, Universidade Estadual do Ceará, Avenida Paranjana 1700, 60000-001 Fortaleza, CE, Brazil; ^4^Departamento de Farmácia, Faculdade de Farmácia, UFC, Rua Capitão Francisco Pedro 1210, 60430-370 Fortaleza, CE, Brazil

## Abstract

The aim of the present work was to evaluate antileishmanial activity of *Astronium fraxinifolium* and *Plectranthus amboinicus*. For the in vitro tests, essential oil of *P. amboinicus* (OEPA) and ethanolic extracts from *A. fraxinifolium* (EEAF) were incubated with 10^6^  promastigotes of *L. (Viannia) braziliensis*. The OEPA was able to reduce the parasite growth after 48 h; nonetheless, all the EEAFs could totally abolish the parasite growth. For the in vivo studies, BALB/c mice were infected subcutaneously (s.c.) with 10^7^ *L. braziliensis* promastigotes. Treatment was done by administering OEPA intralesionally (i.l.) for 14 days. No difference was found in lesion thickness when those animals were compared with the untreated animals. Further, golden hamsters were infected s.c. with 10^6^ *L. braziliensis* promastigotes. The first protocol of treatment consisted of ethanolic leaf extract from *A. fraxinifolium* (ELEAF) administered i.l. for 4 days and a booster dose at the 7th day. The animals showed a significant reduction of lesion thickness in the 6th week, but it was not comparable to the animals treated with Glucantime. The second protocol consisted of 15 daily intralesional injections. The profiles of lesion thickness were similar to the standard treatment. In conclusion, in vivo studies showed a high efficacy when the infected animals were intralesionally treated with leaf ethanolic extract from *A. fraxinifolium*.

## 1. Introduction

Leishmaniasis is a disease that affects human beings and animals and is caused by the protozoa parasite of the genus* Leishmania* which is transmitted by the bite of infected female phlebotomine sandflies and displays a spectrum of manifestations which goes from cutaneous involvement (CL) with late destruction of mucous membranes to generalized systemic visceral disease (VL) with fatal outcome if not treated [1]. According to the World of Health Organization [2], about 0.2 to 0.4 million of new VL cases and 0.7 to 1.2 million of CL occur in the world. Brazil and other nine countries, mostly situated in Africa and South America, are responsible for about 70 to 75% of global incidence of the disease [3]. Brazil has reported 23,793 CLcases in 2012 [4]. Various factors contribute to the increase of the disease incidence, for instance, the process of urbanization which alters the environment in decurrence of economic and social pressures [5]. The main* Leishmania* species which cause the CL disease in Brazil are* L. (Viannia) braziliensis*,* L. (Leishmania) amazonensis*, and* L. (V.) guyanensis* [6].* L. (V.) braziliensis *is the most common aetiologic agent of CL disease in Brazil and other countries in Latin America, and it can be found in endemic zones from the different regions of Brazil [6]. American cutaneous leishmaniasis is a form of disease that causes a single or various cutaneous lesions that can heal spontaneously. Nonetheless, in some cases when mucosa, such as nasal or oral mucosa, is injured, treatment is necessary; otherwise, permanent sequelae may occur [7]. The clinical form and severity of the disease may depend on the* Leishmania* species [8] and/or to the individual immune response [7]. HIV-positive individuals are probably at high risk of developing cutaneous leishmaniasis [9] probably at more severe forms of the disease. This reveals a new challenge in terms of treatment of the disease in immunocompromised individuals. Early diagnosis and treatment are important to prevent sequelae. Pentavalent antimonials are still the treatment of choice for over 50 years; however, they comprise several problems, such as endovenous use and prolonged and high-cost treatment [10]. Increased incidence of resistance to the drug has been described [8]. Another problem is related to the lower efficacy of the drugs in children compared to adults [11]. Alternatively, pentamidine and amphotericin B may be used. In general, these compounds are also expensive and require long-term treatment [8]. Another point to take into account is that the drugs available for CL treatment produce significant side effects due to their high toxicity and tissue drug accumulation, which includes myalgias, nausea, vomiting, cardiac arrhythmia, hepatitis, or pancreatitis [12, 13]. Miltefosine and fluconazole have recently showed effectiveness showed effectiveness against CL [14, 15], but despite their lower toxicity, these second line drugs are not useful against other forms of leishmaniasis [13]. Furthermore, the continuous use of ineffective drugs has led to the development of resistance to their compounds [16], which have stirred an urgent need for novel, effective, and safe drugs for treatment of leishmaniasis. Several studies have been done in order to evaluate antileishmanicidal activity of medicinal plants [17, 18]. The species* Astronium fraxinifolium* Schott, popularly known as “gonçalo alves,” occurs in the tropical savannas of central Brazil (Cerrado Brasileiro), in soils with good fertility, and it can also be found in the northeastern region of the country. The* Myracrodruon urundeuva *species, a plant of the same family, native in northeastern Brazil, is widely used in folk medicine for treatment of various dermatological disorders and is known to have antimicrobial [19], antiulcer [20], and anti-inflammatory activities [21].* Plectranthus amboinicus*, popularly known as malvarisco, a native aromatic plant of India and cultivated in many parts of the world, including Brazil, belongs to the Lamiaceae family and the genus* Plectranthus*. It is popularly used in the treatment of various diseases including skin, digestive tracts, urogenital, respiratory disorders, infections, and pain [22]. Several studies have been published confirming much of the popular use of* Plectranthus amboinicus*, such as anti-inflammatory and antimicrobial activities [23–27]. The aim of the present work was to demonstrate in vitro and in vivo antileishmanial activity of* Astronium fraxinifolium *and* Plectranthus amboinicus*.

## 2. Methods

### 2.1. Parasites

The strain of* L. (V.) braziliensis* (MHOM-BR-94-H3227) was grown in Schneider culture medium (Sigma Chem. Co., St. Louis, Mo, USA), containing 10% heat-inactivated foetal bovine serum (Sigma), 200 U penicillin/mL, and 200 mg streptomycin/*μ*L (Sigma). The strain of* L. braziliensis* (MHOM-BR-94-H3227) was isolated from a patient with cutaneous leishmaniasis and characterized by polymerase chain technique and by monoclonal antibodies [28].

### 2.2. Plant Materials

The ethanolic extracts of the bark, sapwood, and leaves of* A. fraxinifolium* were collected in the city of Lavras da Mangabeira (06°45′12′′S 38°57′52W 239 m), Ceara, Brazil. The extraction was obtained by Gonçalves Jr. and described elsewhere (Gonçalves Jr. et al., manuscript in preparation). All the extracts contained phenols and tannins. The essential oil of* P. amboinicus* was provided by Braga, MA, and description of the protocol was done according to Gonçalves et al., 2012 [26]. The major component of the essential oil was carvacrol.

### 2.3. In Vitro Studies

#### 2.3.1. Antileishmanial Evaluation

The in vitro evaluation of the antileishmanial activity of the essential oil from* P. amboinicus* and of ethanolic extracts (bark, sapwood, and leaf) from* A. fraxinifolium* was performed in 48-well microplates. Each well received 200 *μ*L of medium containing 10^6^ promastigotes of* L. braziliensis*, 200 *μ*L Schneider supplemented medium, and 2.5% essential oil of* P. amboinicus*. Ethanolic extracts from* A. fraxinifolium* were added at 2.5 mg/mL concentrations in 1% dimethyl sulfoxide or DMSO (VETEC, Brazil) to the wells. The plates were left at 26°C in a BOD incubator during 24, 48, and 72 h, without shaking. Then, the parasites were fixed with 2% formalin, stained with Trypan blue, and visualized using a light microscope at 400x magnification. The parasites were counted in a Neubauer chamber. As controls, 1% DMSO and amphotericin B (Sigma, USA) were used.

#### 2.3.2. Cytotoxicity Assay

Macrophage cell line RAW 264.7, obtained from the Rio de Janeiro Cell Bank (BCRJ, Brazil), was grown in microculture plates containing Minimum Essential Media medium supplemented with 10% v/v foetal bovine serum, penicillin (100 U/mL), and streptomycin (100 *μ*g/mL). Cells were incubated at 37°C in a humidified 5% CO_2_ atmosphere. Before each experiment, the cells were incubated in medium without foetal calf serum for 24 h to obtain cells in the G_0_ phase of the cell cycle. For each experiment, cells were removed from the culture medium and incubated with 0.25% trypsin and 0.02% ethylenediaminetetraacetic acid (v/v) for approximately 10 min at 37°C. Trypsin was inactivated by adding the same volume of medium containing foetal bovine serum. The suspension was centrifuged for 10 min at 1500 ×g. The supernatant was discarded, and the cells were resuspended in culture medium. The macrophages were quantified using a Neubauer chamber and subcultured (1 × 10^5^ cells/mL) into a 96-well microplate for 24 h. The essential oil of* P. amboinicus* was tested from 0.125 to 4.0% v/v and the ethanolic extracts of* A. fraxinifolium* from 0.039 to 2.5 mg/mL concentrations. After an incubation of 24 h, 100 *μ*L of the supernatant was discarded and 100 *μ*L of 3-(4,5-dimethylthiazol-2-yl)-2,5-diphenyltetrazolium bromide (MTT) at 500 *μ*g/mL dissolved in phosphate-buffered saline (PBS), pH 7.4, was added to the wells after incubation for 4 h at 37°C. Thereafter, 10% sodium dodecyl sulphate in 0.01 N HCl was added to solubilise the formazan crystals [29]. Plates were then incubated for 17 h, and readings were performed at 570 nm using a microplate reader. Assays were performed in triplicate.

### 2.4. In Vivo Studies

#### 2.4.1. Animals

Three- to four-month adult male golden hamsters (*Mesocricetus auratus*) and BALB/c, male, 8 weeks, obtained from the Central Animal Facility of the Department of Pathology and Legal Medicine of the Federal University of Ceará, were housed in groups of six to eight per cage with free access to water and food. All procedures involving the uses of animals were approved by the Ethics Committee for Animal Research of the Federal University of Ceará (protocol number 75/2011).

#### 2.4.2. Treatment with the Essential Oil of* P. amboinicus*

BALB/c mice were divided into 3 groups (5 animals per group), untreated, Glucantime, and test. The animals were injected subcutaneously in right hind footpad with 10^7^ stationary phase* L. braziliensis* promastigotes in 20 *μ*L of sterile saline. Lesion sizes were measured weekly with a dial gauge caliper (0.01 mm sensitivity, Mitutoyo, Japan) and expressed as the difference between the thicknesses (mm) of the infected and contralateral uninfected footpads.

The treatment was initiated when the lesions appeared (about the 27th day). Dosis of 20 *μ*L of 5% essential oil of* P. amboinicus* in 1% DMSO was administered intralesionally once a day for 14 days. Meglumine antimoniate (Glucantime), injected intramuscularly at the dose of 60 mg/kg/day, 20 *μ*L, was used as reference drug. Untreated animals were intralesionally injected with 1% DMSO. The uninfected contralateral footpad was also intralesionally injected with the same solution used for treatment. This procedure was done in order to check toxicity of the material used to treat the animals.

#### 2.4.3. Treatment with Ethanolic Extracts of Leaves from* A. fraxinifolium*

Golden hamsters, weighing about 70 g, were divided into 8 groups with 5 animals per group (2 untreated, 2 Glucantime, 2 tests, 2 uninfected). The animals were injected subcutaneously in the right hind footpad with 10^6^ stationary phase* L. braziliensis* promastigotes in 20 *μ*L of sterile saline. Lesion sizes were measured weekly with a dial gauge caliper (0.01 mm sensitivity, Mitutoyo, Japan) and expressed as the difference between the thicknesses (mm) of the infected and contralateral uninfected footpads. The treatment was initiated when the lesions appeared (about the 20th day after infection). Meglumine antimoniate (Glucantime), injected intramuscularly at the dose of 60 mg/kg/day, 20 *μ*L, was used as reference drug. Untreated animals were intralesionally injected with 1% DMSO. For the test treatment, two protocols were used. The first treatment protocol consisted of 20 *μ*L ethanolic leaf extract (at 2.5 mg/mL in 1% DMSO) administered intralesionally for 4 consecutive days. At the 7th day, a single booster (40 *μ*L) was given. The second treatment protocol consisted of intralesional injections of 20 *μ*L ethanolic leaf extract administered for 15 consecutive days. The animals were also weighed during this period. The animals were euthanized after the 8th week of infection.

### 2.5. Parasite Load

The number of parasites in the lesions was quantified by the limiting dilution technique as previously described [30]. Briefly, after the treatment, the animals were euthanized by inhalation of halothane (Sigma-Aldrich) and submerged in 3% iodized alcohol up to 3 minutes to allow decontamination. The infected footpads were removed aseptically and macerated in a Petri dish with 2 mL of Schneider medium and left to stand for 5 minutes. After homogenization, the material was tenfold serially diluted in Schneider medium supplement with 10% foetal bovine serum. One hundred microliters of these dilutions was distributed into 96-well flat bottom plates containing agar-blood in 6 replicates per concentration. The plates were incubated at 25°C and observed under an inverted microscope (Nikkon, Japan) every 3 days, up to a maximum of 30 days, to record the dilutions containing promastigotes. The final number of parasites per tissue was determined using the ELIDA software version 12c [31].

#### 2.5.1. Statistical Analysis

The data relating to the parasite load were analyzed using the nonparametric Mann-Whitney test. The lesion sizes from treated and untreated animals were analysed by the one-way Anova and complemented by the Tukey-Kramer test for multiple comparisons. The analyses were performed using GraphPad Prism version 5.0 and the GraphPad InStat version 3.01 programs. The level of significance for a null hypothesis was 5% (*P* < 0.05).

## 3. Results and Discussion

In the present study we decided to evaluate two medicinal plants,* Plectranthus amboinicus* and* Astronium fraxinifolium*.* P. amboinicus* is original from India, but it has easily been adapted to the conditions of soil and climate of Brazilian northeastern region. As it was shown in our previous study [26], the major component in essential oil obtained from* P. amboinicus* was carvacrol (yield above 90%). Carvacrol is a monoterpene phenol, isomer to thymol, and presents various biological activities such as antimicrobial, antitumor, and anti-inflammatory activities [32].* Astronium fraxinifolium* is original from the central-west region of Brazil, but it has also been found in various regions of the northeastern region of the country. Its activities are poorly known unlike the other plant from the same family,* M. urundeuva*, which is original from the northeastern region of Brazil. As* A. fraxinifolium* can be cultivable, it is not at extinction risk.

**Figure 1:**
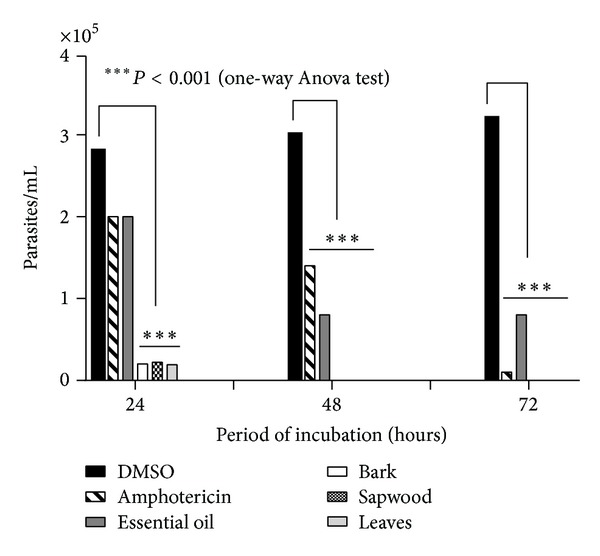
In vitro antileishmanial activity of essential oil of* P. amboinicus* at 2.5% in 1% DMSO and ethanolic extracts (from bark, stem bark, and leaf) of* A. fraxinifolium* at 2.5 mg/mL incubated with 10^6^ promastigotes of* L. braziliensis* during 24 h, 48 h, and 72 h. The parasites were fixed in formaldehyde, stained with Trypan blue and visualized at light microscope at 400x magnification.

As it was shown in Figure 1, essential oil of* P. amboinicus* at 2.5% was able to reduce the viability of* L. braziliensis *promastigotes in the first 48 h, similar to the reference drug (*P* < 0.001) when compared to parasites incubated with 1% DMSO. However, at 72 h, no significant decrease was observed in comparison to the previous period of 48 h. On the other hand, all the ethanolic extracts (from bark, sapwood, and leaves) from* A. fraxinifolium* at 2.5 mg/mL totally abolished the parasite growth after 48 h. In vitro results showed that both essential oil of* P. amboinicus* (EOPA) and ethanolic extracts from* A. fraxinifolium *(EEAF) presented potential activity against* L. braziliensis *promastigotes. However, EOPA did not cause 100% mortality in parasite when compared to EEAF. Nonetheless, we decided to test the EOPA treatment in animal models based on data from other researchers [33] who showed a good antileishmanial activity of essential oil, in the case of* Lippia sidoides* Cham., which contained 43.7% of carvacrol.

**Figure 2:**
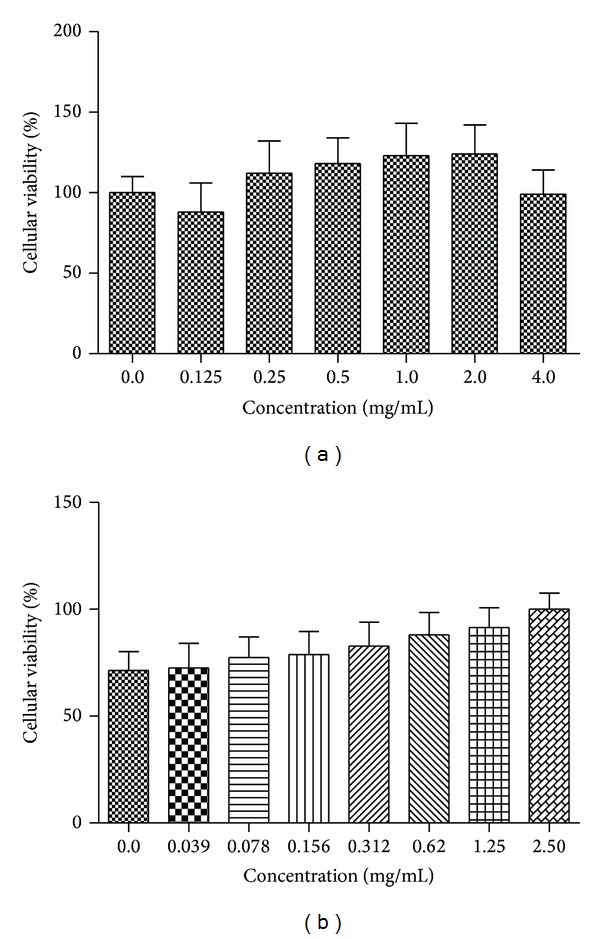
Cytotoxicity of the plant material. Percentage of cellular viability (mean ± standard error mean) using RAW 264.7 macrophages after 24 h incubation with essential oil of* P. amboinicus* (a) or with ethanolic leaf extract of* A. fraxinifolium* (b) at various concentrations.

No cytotoxic activity of the plant materials was observed when they were incubated in different concentrations with RAW 267.4 macrophage at 24 hours (Figure 2).

**Figure 3:**
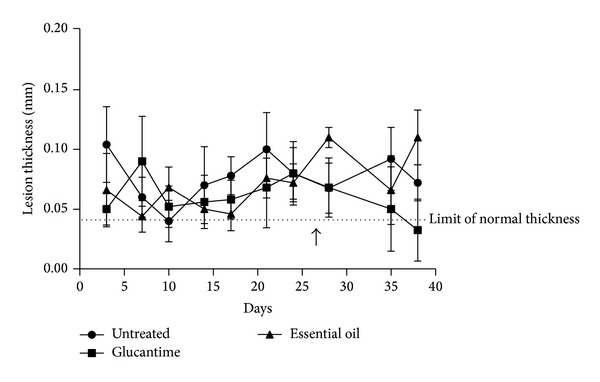
Lesion thickness of BALB/c mouse footpad infected with 10^7^ promastigotes of* Leishmania braziliensis* and treated with essential oil of* Plectranthus amboinicus*. The protocol of treatment was 15 daily doses of 20 *μ*L of 2.5% plant material. The arrow indicates start of treatment (about the 27th day).

Despite the promising in vitro results, in vivo experiments did not reveal a good efficacy of the treatment with EOPA in mouse model. No difference was found when the animals treated with the plant material and the untreated animals were compared in terms of lesion thickness (Figure 3). This fact could be related to the concentration of the essential oil, to the route of administration, or even to the period of treatment. Probably the essential oil of* P. amboinicus* should be associated with other plant materials in order to improve the efficacy of treatment. We have previously observed through in vitro experiments that the association of EEAF with EOPA led to total inhibition of parasite growth after 48 h (data not shown). Monzote et al. [17] have demonstrated that essential oil from* Chenopodium ambrosioides *showed a synergic activity with pentamidine against* Leishmania amazonensis* promastigotes. In respect to the route of administration, Patrício et al. [34] have shown that the treatment administered via the intralesional route was more effective than the oral route. According to their data, mice infected with* L. amazonensis* and treated with the hydroalcoholic crude extract from the leaves of* Chenopodium ambrosioides* administered via the intralesional route showed a greater reduction of the parasite load in various organs compared to the other route of administration.

Additionally, we decided to use the gold hamster model to evaluate the activity of our plant materials in vivo based on the fact that hamsters are known to be highly susceptible to* Leishmania* subgenus* Viannia* infection [35]. Also, male animals were tested because this gender is more susceptible to the* Leishmania* infection than the females, and this susceptibility seems to be related to the type of cytokine produced [35].

**Figure 4:**
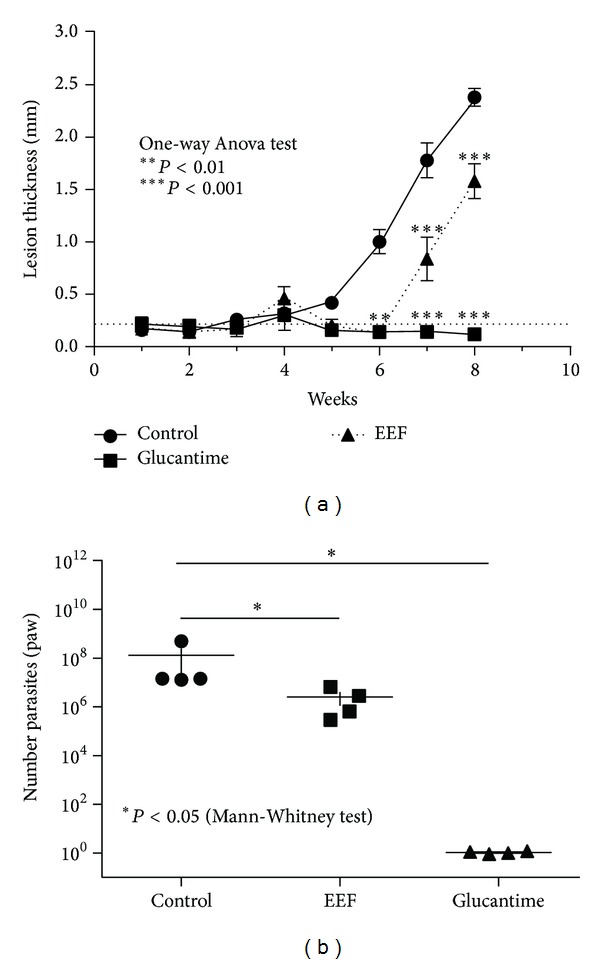
Lesion thickness (a) and number of parasites (b) in hamster footpad infected with 10^6^ promastigotes of* Leishmania braziliensis*. The animals were treated with leaf ethanolic extract from* Astronium fraxinifolium* (EEF). The protocol of treatment was 4 dairy doses of 20 *μ*L of 2.5 mg/mL plant material and 1 booster dose of 40 *μ*L of the plant. The treatment started when the lesions appeared (3 weeks).

Hamsters treated with 4 daily doses of ethanolic leaf extract from* A. fraxinifolium* and 1 booster dose showed a significant reduction of lesion thickness after the 6th week after infection (*P* < 0.001) in comparison to the untreated animals, but it was not comparable to the reduction observed in the group of animals treated with Glucantime (Figure 4(a)). In respect to parasite growth, the animals treated with the plant material showed lower parasites in the infected footpad after treatment in comparison to the untreated animals (Figure 4(b), *P* < 0.05). However, a higher number of parasites were found when the group of animals treated with the plant material was compared with the group treated with Glucantime.

**Figure 5:**
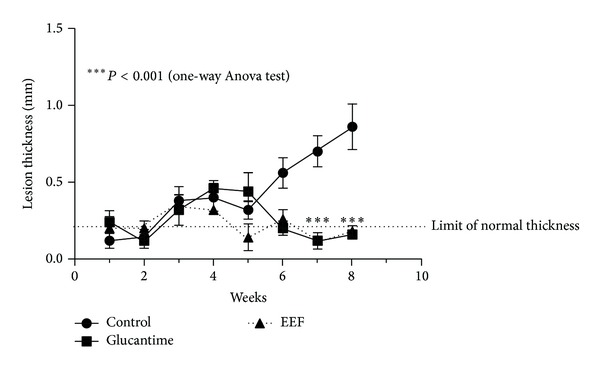
Lesion thickness of hamster footpad infected with 10^6^ promastigotes of* Leishmania braziliensis*. The animals were treated with leaf ethanolic extract from* Astronium fraxinifolium* (EEF). The protocol of treatment was 15 dairy doses of 20 *μ*L of 2.5 mg/mL plant material. The treatment started when the lesions appeared (3 weeks).

Hamsters treated with 15 daily doses of the plant material showed a profile of lesion thickness similar to those treated with Glucantime, much lower than that observed in the untreated animals (Figure 5, *P* < 0.001). After the 7th week of infection, the lesion thickness in the group treated with the plant material was below the limit of normal thickness. Any loss of weight was not observed in the animals treated with the plant material. No inflammation was found in the uninfected animals treated with the plant material.

In conclusion, both essential oil from* P. amboinicus* and ethanolic extracts from* A. fraxinifolium* were able to reduce parasite growth in vitro; however, studies in vivo showed a much higher efficacy when hamsters were intralesionally treated with leaf ethanolic extract from* A. fraxinifolium*. More studies with these plant materials are necessary to demonstrate the efficacy of the treatment administered in association with other plant materials and/or the reference drug.
